# Fast and Scalable Private Genotype Imputation Using Machine Learning and Partially Homomorphic Encryption

**DOI:** 10.1109/access.2021.3093005

**Published:** 2021-06-28

**Authors:** ESHA SARKAR, EDUARDO CHIELLE, GAMZE GÜRSOY, OLEG MAZONKA, MARK GERSTEIN, MICHAIL MANIATAKOS

**Affiliations:** 1 Tandon School of Engineering, New York University, New York, NY 11201, USA; 2 New York University Abu Dhabi, Abu Dhabi, United Arab Emirates; 3 Program in Computational Biology and Bioinformatics, Yale University, New Haven, CT 06520, USA

**Keywords:** Genotype imputation, machine learning, privacy-preserving computation

## Abstract

The recent advances in genome sequencing technologies provide unprecedented opportunities to understand the relationship between human genetic variation and diseases. However, genotyping whole genomes from a large cohort of individuals is still cost prohibitive. Imputation methods to predict genotypes of missing genetic variants are widely used, especially for genome-wide association studies. Accurate genotype imputation requires complex statistical methods. Due to the data and computing-intensive nature of the problem, imputation is increasingly outsourced, raising serious privacy concerns. In this work, we investigate solutions for fast, scalable, and accurate privacy-preserving genotype imputation using Machine Learning (ML) and a standardized homomorphic encryption scheme, Paillier cryptosystem. ML-based privacy-preserving inference has been largely optimized for computation-heavy non-linear functions in a single-output multi-class classification setting. However, having a large number of multi-class outputs per genome per individual calls for further optimizations and/or approximations specific to this application. Here we explore the effectiveness of linear models for genotype imputation to convert them to privacy-preserving equivalents using standardized homomorphic encryption schemes. Our results show that performance of our privacy-preserving genotype imputation method is equivalent to the state-of-the-art plaintext solutions, achieving up to 99% micro area under curve score, even on real-world large-scale datasets up to 80,000 targets.

## I. INTRODUCTION

Large-scale Genome-Wide Association Studies (GWAS) have tremendous value in understanding the relationship between genetic loci and disease risk and heritable traits [1]. Understanding the genotypic landscape of millions of diverse individuals is essential for characterizing and investigating rare diseases and genotype-phenotype associations. Genotype imputation methods predict the genotypes of missing Single Nucleotide Polymorphisms (SNPs) by taking advantage of the high correlation between SNPs in haplotype blocks; hence, they provide opportunities for sequencing a larger number of individuals through cheaper sequencing techniques [2].

Sharing and analysis of genomics data is challenging due to the size of the data, which can be sometimes in the order of petabytes [3]. Genetic information is being increasingly clinically relevant and used for personalized medicine requiring hospitals to perform analysis and calculations of genomes [4], [5]. Therefore, the entities which require computationally heavy genomic analysis, do not generally have the resources to perform it locally, and commonly outsource it to the cloud. For example, Stanford Center for Genomics and Personalized Medicine, computes on genomic data using Google Cloud and Google Big Query [6]. Real-world genotype imputation requires thousands of genotypes to be predicted in real time and is one of the many genomic analyses that can be outsourced to the cloud [7], [8]. Having an outsourced entity for such computations also helps in maintenance of the prediction models. As the size of the genomic databases increases (for example, when more genomes are sequenced from individuals with different backgrounds and ancestries), the predictive models can be updated and patched directly in the cloud. With the increasing wealth of genetic information available to train, the outsourced models reach unprecedented accuracy on test data, even when predicting a large number of outcomes [3]. However, to use these outsourced models for inference, the sensitive data must be sent and computed on the cloud. Since the genetic information of these individuals (who want to use the *trained* outsourced genetic analysis model) is sensitive and prone to re-identification for malicious purposes, outsourcing such analyses introduces serious privacy concerns. For example, legislation like the General Data Protection Regulation (GDPR) [9] in the European Union may prohibit outsourcing calculations on sensitive data even when the patients consent to share their genetic information. Therefore, sensitive data must be protected not only from the attackers who try to snoop in network communications or try to breach genomic databases in the cloud, but also from the cloud itself. In a nutshell, any privacy-preserving genotype imputation must guarantee the protection of the sensitive data while imputing thousands of genotypes in real-time.

### A. RELATED WORK

Current genotype imputation studies are based on the hypothesis that short genomic regions in a population of individuals cluster into groups of similar regions as we inherit many of our genomic regions from our ancestors [2]. There are several available programs for genotype imputation. The most commonly used software, IMPUTE2 [10] and Beagle [11], are different implementations of hidden Markov models to observe the unknown genotypes using training data from a population of individuals. These software take a set of known SNP genotypes (also called tag SNPs) as input and predict the genotypes of unknown SNPs (also called target SNPs). As the amount of data increased, the aforementioned models focused on maintaining high accuracy. Imputation in the encrypted domain, however, remains a challenge due to high computational overheads.

Several methodologies have been proposed for ensuring the privacy of outsourced data. Among them, multi-party computation, functional encryption, and homomorphic encryption are prime candidates for data protection, with different properties and intended usage [12]. Multi-party computation has been used in genome wide association studies [13] but is network-bound, assuming constant interaction between the participating nodes. But it is possible to leak information when multiple parties collude. This vulnerability makes it unsuitable for sensitive applications like genotype imputation. Functional encryption, on the other hand, can operate using encrypted data directly. Still, the data has to be encrypted for a specific algorithm, and further algorithm modifications are not allowed without re-encryption.

The privacy solutions mentioned above consider different threat models comprising of a malicious end-user (trying to infer about cloud models), man-in-the-middle attacks (leveraging side-channels), or malicious cloud (trying to infer about client data) or a combination thereof [14]. In our work, we consider an honest but a curious attacker. In that scenario, imputation using homomorphic encryption is a promising privacy-preserving solution, since the operands and the outputs do not need to be decrypted during rest, transit or use. Fully Homomorphic Encryption (FHE) requires only a single time encryption and applications developed with FHE can be used infinite times without re-encryption. FHE has been used for medical data analysis such as on studies related to the analysis of Electro Cardiogram (ECG) [15] and on diabetes and heart disease studies [16]. While FHE is algorithmically very powerful and is quantum resistant, it incurs prohibitive performance overhead when implemented without optimizations. Therefore, to make privacy-preserving solutions based on FHE faster, batching is used. Partially Homomorphic Encryption (PHE), on the other hand, allows unlimited manipulation of data and may be scaled, unlike FHE that requires refreshing the ciphertexts if pre-defined computational bounds are exceeded. The main drawback of PHE schemes is the limit in the types of algorithms it can express.

The Paillier cryptosystem is a prominent example of PHE [17]. Paillier’s native operation on encrypted data is a modular multiplication of operands of a few thousand bits, while FHE schemes based on the Ring Learning With Errors (RLWE) problem add/multiply polynomials of a few thousand degrees and few hundred bits coefficients. Consequently, operating on PHE ciphertexts is inherently faster than FHE ciphertexts. In order to accelerate algorithms on FHE, batching is used. This implies that computation should be easily parallelizable and without dependencies. PHE schemes do not rely on batching for performance and this may serve as another advantage in genotype imputation since we can potentially impute any number of individuals (performance is not tied to the batch size). Moreover, algorithms implemented using Paillier may be further accelerated using hardware accelerators like CoPHEE [18]. Currently, no dedicated ASIC accelerator exists for FHE schemes, even though there is some work-in-progress in FPGA-and GPU-based acceleration. A major difference between PHE and FHE is that Paillier is an accepted ISO standard (ISO/IEC 18033-6:2019, part 6) [19]. Due to their young age of RLWE-based schemes, no such standard exists and are currently under the process of standardization. While there is a trade-off between security guarantees, standardization, performance, and acceleration capabilities between FHE schemes and Paillier, for a private genotype imputation software, we choose Paillier for its advantages in the context of healthcare. Since the healthcare industry is heavily regulated, FHE-based solutions may take a few more years before they can be used in practice.

### B. OUR APPROACH

**FIGURE 1.**
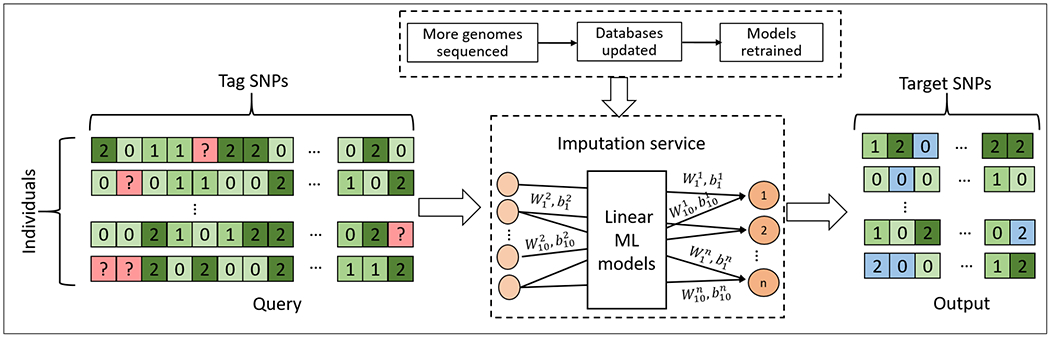
Genotype imputation as a service. The machine learning model is trained using publicly available genomes (e.g. those from 1000 genomes project). The ML-based imputation model takes the tag SNP genotypes and imputes the target SNP genotypes. The ML model is a combination of N linear models where N is the number of target SNPs being imputed. The model uses that top 10 tag SNP genotypes as features based on the mutual information between target and tag SNP gentotypes. The weights and biases, written as *W*^*n*^_*m*_ and *b*^*n*^_*m*_, are represented by connections where the superscript, *n*, is the target SNP and the best correlated tag SNP is represented by *m*. The model can be continuously updated as the training database is updated with more sequenced genomes.

**FIGURE 2.**
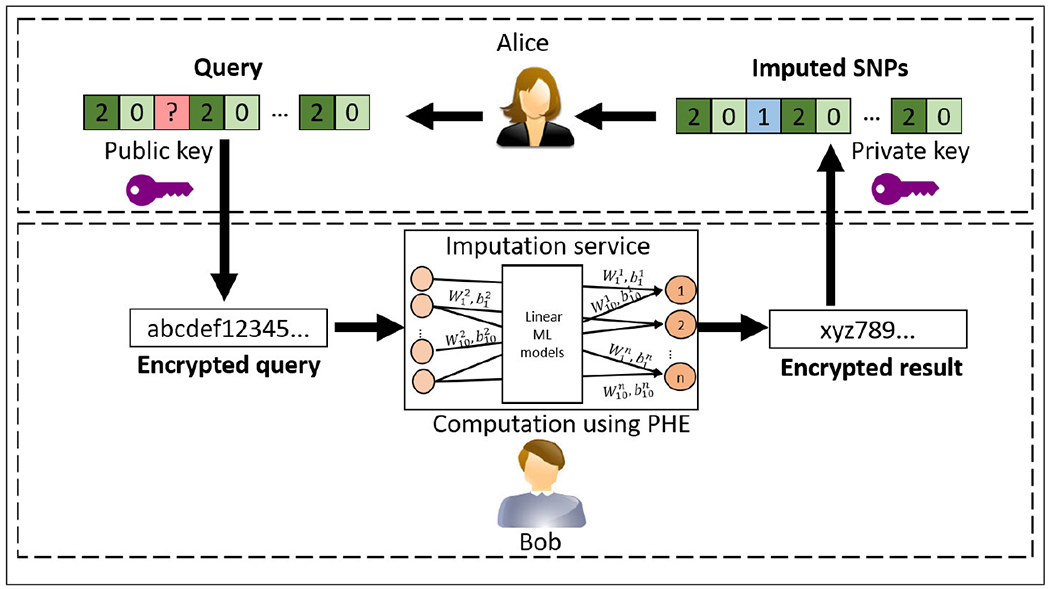
Encryption of tag SNP genotypes and decryption of target SNP genotypes. The figure shows Alice, who wants to perform genotype imputation on her genome. She encrypts her tag SNP genotypes using a public key and sends it to the untrusted cloud-based imputation service maintained by Bob. Bob maintains the imputation service by training it with new data when available according to Fig. 1. Bob performs imputation and sends the encrypted result back to Alice. Since Bob does not possess the private key and the data is not decrypted during computation, the sensitive data remains a secret through the entire process.

In this work we explore the possibility of using standardized encryption technique for imputation task for thousands of genotypes. But traditional imputation techniques need non-linear function support not provided by PHE, therefore we explore for the best-performing models which could be re-purposed for imputation. Recently, Machine Learning (ML) has seen an unparalleled growth in usage in genomics because of its precision in classification, regression, and sequence prediction tasks. Genotype imputation, can also be performed using ML-based models, and be a part of Machine Learning as a Service (MLaaS) ecosystem. Fig. 1 describes genotype imputation, where an ML model is used to impute the missing genotypes. This figure depicts the common supply chain of imputation as a service, in which training does not have to be in the encrypted domain. The training of the imputation models use the publicly available data from individuals who have consented to the usage of their data for analysis and thus, can be performed in plaintext. The model is continuously improved as more training data (i.e. more reference panels) become available. The encryption-decryption processes of tag and target SNPs are explained in detail in Fig. 2. The cloud, represented by Bob, offers imputation as a service and honestly imputes and predicts target SNPs when required by the user, represented by Alice. But Bob may get curious about the data (and thus, individuals and their genetic traits) being analyzed and therefore, is untrusted and cannot be given tag SNP genotypes of an individual in plaintext. Users encrypt their queries (i.e tag SNP genotypes) with a public key and send them to the server. The server then uses the trained imputation model to perform imputation on the encrypted query and sends back the encrypted results to the users. In our privacy-preserving solution, only the data-owner, Alice, can see the sensitive data in plaintext.

ML algorithms for prediction problems have several non-linear operations like exponentiation, activation, or feedback. Since Paillier, although a standardized algorithm for private computation, can only implement a single type of operation, it cannot readily support such complex ML-models. In this study, we combine the genotype imputation accuracy offered by machine learning models with the efficiency and maturity of PHE to enable privacy in outsourcing genotype imputation. To this end, we develop suitable linear ML-models that maintain the high accuracy of traditional ML algorithms and port them to the encrypted domain using PHE. We address various incompatibility issues, such as the lack of floating-point support and negative numbers in PHE, and develop a novel methodology for fast and scalable privacy-preserving genotype imputation.

Our contributions can be summarized as follows:

- We design linear ML models having similar accuracy and Micro Area Under Curve (MAUC) metrics compared to commonly used non-linear techniques for genotype imputation state-of-the-art techniques [10], [11].
- To the best of our knowledge, this is the first private imputation technique based on a standardized homomorphic encryption scheme. We fine-tune the models to facilitate their privacy-preserving implementation using PHE. We introduce several optimizations in traditional PHE schemes catering to the fast data-intensive private sequence prediction for genotype imputation.
- We implement privacy-preserving non-linear machine learning models along with the non-linear functions in the encrypted domain (using FHE) as a proof-of-concept to showcase that private imputation solutions without approximations in the model or optimizations during private computation may lead to prohibitive overheads.
- We further test our models using two independent datasets: Individuals from Genotype Tissue Expression (GTEx) [20] and from Avon Longitudinal Study of Parents and Children (ALSPAC) [21] to ensure scalability of our approach.

The rest of the paper is organized as follows: Section II discusses the background on plaintext genotype imputation, threat model, machine learning, and the performance metrics. We discuss our methodology in Section III. Experimental results presented in Section IV compare our methodology with state-of-the-art methods and more complex non-linear models in terms of accuracy and computation cost. Finally we discuss the important takeaways in Section V and conclude in Section VI.

## II. PRELIMINARIES

### A. GENOTYPE IMPUTATION

Genotype imputation is the process of predicting the genotypes (i.e 0 for existence of reference allele in both haplotypes; 1 for existence of one reference and one alternative allele; 2 for existence of alternative allele in both haplotypes) of SNPs in a genome. Genotype imputation is performed by using the information that the SNPs in a genome are in linkage disequilibrium due to the haplotype structures [2]. The correlations between the SNPs can be inferred using a database of fully characterized genomes such as 1000 Genomes dataset [22]. SNPs in a genome can be classified as tag and target SNPs. Tag SNPs are the ones that can be observed experimentally and target SNPs are in correlation with the tag SNPs and can be imputed computationally. Traditional genotype imputation methods require phasing of the genome into haplotypes [10], [11]. Here we propose a privacy-preserving machine learning based imputation method, that takes the tag SNP genotypes as features and predicts the target SNP genotypes using partially homomorphic encryption and without the need for phasing.

### B. THREAT MODEL

We consider an *honest but curious* imputation server similar to the threat models in genome privacy literature [23]. We assume that the cloud gathers a *training dataset* that comprises of reference panels from different individuals who have agreed to share their data for further analysis and for building imputation models. This dataset is sent to the cloud in plaintext and training at the cloud also happens in plaintext. As more and more genomes are sequenced, more and more reference panels become available for the server to train or update the existing imputation models [7]. The server uses these reference panels to train models to impute genomic sequences, i.e. predict the genotypes of target variants given the genotypes of tag variants. This part of our threat model is common in genotype imputation supply chain for publicly available imputation servers where computation happens in plaintext [7], [8]. The training required for building imputation as a service is depicted in Fig. 1.

After the imputation model is trained and deployed, individuals, research institutes or hospitals can send tag variants to impute their target variant genotypes. Please note that these tag variants are essentially the *test dataset* and are from individuals who do not want to share their data. However, if the tag variants are sent, stored or analyzed in plaintext, there may be severe privacy risks involved. Privacy of genomic data is different from generic data privacy because 1) genomes are unique to individuals and are extremely identifying and characterizing, even for a partial leak, 2) the impact of data leakage is permanent as, unlike passwords, genome of an individual cannot be changed, 3) genomic data of one individual may lead to information about their direct relatives. Therefore, although a cloud may impute honestly, but still has the incentive to be curious and can achieve stealthy and substantial malicious objectives using the genomic data. Moreover, under local or institutional privacy guidelines, the cloud might have to be mandated to protect the healthcare related data and therefore services that handle genomic data have to incorporate access control, execution in trusted platforms, and encrypted storage to thwart external attackers [6], [7]. In our threat model we consider the cloud to be an honest but a curious entity which may misuse the data while computing on it honestly. In this scenario, the sensitive data must remain encrypted during rest, transit, and also during computation. Fig. 2 describes private imputation in our threat model where the imputation occurs on encrypted data using homomorphic encryption. As the tag and target variants always remain encrypted, this also protects against external attackers trying to cause breach in databases.

### C. PREDICTION OF LINEAR AND NON-LINEAR ML MODELS

Machine learning algorithms make various transformations on the input data such that the error (loss) between the predicted output and actual output is minimized. For classification problems, the transformations are such that the data could be categorized by distinct decision boundaries. For regression problems, the transformations directly predict the real-valued outputs. Linear transformation (affine formally) of the input data *x*: *Wx* + *b*, does not help in classification problems with non-linear decision boundaries present in many real-world problems. Thus, non-linear models like logistic regression, Support Vector Machine, etc. and non-linear functions like Rectified Linear Unit (ReLU), Sigmoid, Tanh, Softmax in Neural Networks have become an integral part of classification problems. The predicted output *O* of a non-linear ML model is defined as *O* = *f (Ax* + *b*) where *f* (.) is a non-linear transformation. A linear model is easier to implement in encrypted domain for complex problems like genotype imputation. While the efficiency of non-linear models are higher, they are extremely hard to implement with homomorphic encryption. Therefore, for private imputation, solutions resort to implementing just the linear part of a model in encrypted domain. In development of linear models too, there are two options, training a non-linear model but using only the linear parts during inference, or training a linear model. We discuss the design of linear ML-models in Section III-A that can be implemented using homomorphic encryption as discussed in subSection II-D.

### D. HE FOR PRIVACY PRESERVING COMPUTATION

Computing on encrypted data is possible by a special type of encryption called Homomorphic Encryption (HE), which allows operating on encrypted data directly without decryption. Different attempts to implement secure homomorphic computation resulted in different mathematical models. Some of them support limited set of operations which are capable of doing computations only for specific tasks. Others support a universal set of operations and are able to compute general functions (e.g. functions which can be represented as combinational circuits), which are called Fully Homomorphic Encryption (FHE) schemes. The former ones, supporting only limited set of operations (called Partially Homomorphic Encryption, PHE), are unable to compute all functions, but, at the same time, they can be simpler and faster.

A very well known example of PHE is the Paillier encryption scheme. In Paillier, the multiplication of ciphertexts modulo *N*^2^ is homomorphic to the addition of plaintexts modulo *N*, where *N* is an encryption parameter. If *N* is a product of two big primes, its factorization is considered hard. Therefore, the security of Paillier cryptosystem depends on Decisional Composite Residuosity Assumption as well as the hardness to factorize *N* [17]. The scheme is defined as:

$$c=E\left(m,r\right)=g^{m}\cdot r^{N}\;\mathrm{mod}\;N^{2}\;D\left(c\right)=L\left(c^{\lambda }\mathrm{mod}N^{2}\right)\cdot \mu \mathrm{mod}\;N$$

where *m* is the message, *r* is the random part of the encryption *E*, (*N*, *g*) is the public key, (λ, *μ*) is the private key, and *L* is a function defined as L(x)=(x−1)/N.

### E. METRICS

The datasets are largely un-balanced and therefore, we use several metrics to analyze performance. The metrics used in this work has been used to design the model as well as to measure the efficiency of the final model and compare it to the state-of-the-art tools.

#### 1) ACCURACY

Accuracy is defined as the ratio of the correct predictions to the total number of samples. It is given by:

$$Accuracy=\frac{TP+TN}{TP+FP+TN+FN}$$

where TP and TN are the True Positive and True Negative rates and the FP and FN are the False Positive and False Negative rates. We use this accuracy as a design objective for our model.

#### 2) MACRO-AVERAGE ACCURACY

$$\begin{array}{l} Macro-average\;accuracy\\ \;=\frac{1}{No\cdot of\cdot \mathrm{var}iants}\\ \;\times \left[\frac{No\cdot of\;true\;imputations\;for\;a\;\mathrm{var}iant}{No\cdot of\;individuals\;for\;the\;\mathrm{var}iant}\right] \end{array}$$

#### 3) MICRO-AREA UNDER CURVE SCORES

We plot Receiver Operating Curves (ROCs) on the final architecture, to compare true positive rate as a function of false positive rate and then, we measure the area under ROC using micro-averaging over all the genotypes for Micro-AUC (MAUC) score. The MAUC score gives a more comprehensive measure of the performance of the predictive model as it does not depend on the test data and thus, has been used for comparing the performance of various classifiers/prediction models [24].

## III. METHODOLOGY

### A. NEURAL NETWORK DESIGN

Our network design is motivated by two factors: imputation efficiency and ease of implementation with PHE. We follow a structured approach to finalize our model. First, we make a neural network using all the tag SNPs, with a direct connection between the input layers and the output sequence to 1) investigate if linear transformations could be used to predict the output target SNPs adequately, and 2) have a reference (baseline) linear prediction model using all tag SNPs. Next, we build a model that uses a subset of features but achieves a similar accuracy. Since we convert the problem to a multi-class problem for several outputs, we represent the data in one-hot encoded format to improve accuracy, i.e. genotypes [0,1,2] are represented as [0,0,1], [0,1,0], and [1,0,0], respectively. We divided the training data into 5 folds and performed k-fold cross-validation to reduce over-fitting. Using all features in 10k and 1k datasets, we achieve a test accuracy of 85.09% and 95.93%, respectively. Next, we perform another feature extraction mechanism, mutual information, on top of neural network training and investigate the number of top features required to reach reference accuracy (from model using all tag SNPs). Mutual information between two random variables is given by

$$I\left(X;Y\right)=\underset{y\in Y}{\sum }\underset{x\in X}{\sum }p\left(x,y\right)\log\left(\frac{p\left(x,y\right)}{p\left(x\right)p\left(y\right)}\right)$$

where *p(x, y)* is the joint probability density function and *p*(*x*) and *p*(*y*) are individual probability density functions. Considering target labels and features, the mutual information score reflects the dependence between a target SNP genotype (a prediction) and a tag SNP genotype (a feature). We rank the tag SNPs according to the mutual information scores for each target SNP and select the top 10 tag SNP genotypes to predict a particular target SNP genotype as the test accuracy saturates for 10 tag SNPs at 84.404% and 95.52% for the 10k and 1k datasets, respectively.

#### 1) FINAL DESIGN

For imputing *t* target SNPs, we build *t* mini-neural networks that connect the top *x* tag SNPs with the corresponding target SNP, since target SNPs are independent of each other. In our design, these independent outputs have their own set of top 10 tag SNP genotypes as features. We then predict genotype probabilities of each target SNP (output) without any non-linear activation. The total number of models would be the total number of target SNPs to be imputed. The final neural network architecture is such that specific (top 10) tag SNPs are connected (through weights and biases) to each target SNP, forming mini-neural networks for each target SNP genotype prediction, as shown in Fig. 1. The weights (*W*) and biases (*b*) are obtained using Adam optimizer with categorical cross-entropy as loss function. We calculate the final probability of a target SNP genotype belonging to class *k* by normalizing across the classes. The formula for finding probabilities is given by:

$$P\left(y=k|x\right)=\frac{{\left(Wx+b\right)}_{k}}{\sum_{l=0}^{2}{\left(Wx+b\right)}_{l}}$$

where *x* is the subset of tag SNPs, which are selected using mutual information. Please note that the probability calculation for each target SNP is not required for prediction of that particular target SNP genotype. The target SNP (position in one-hot encoding) that has relatively the highest probability is the final prediction i.e. the highest amongst the three decrypted values. This approximation of not calculating probabilities and sending the relative values back to the client is another approximation which we incorporated for implementation of private imputation. The probability calculation shown here is used to plot ROC curves to compare performance.

### B. FINE-TUNING NEURAL NETWORK FOR PAILLIER IMPLEMENTATION

In our threat model, we want to protect the data of the user from the untrusted server. The user query *x* is encrypted by the user as *E*(*x*) and can be multiplied with the plaintext weights using Paillier. Encrypted prediction probabilities *E*(*O*) are given by *E*(*x*) × *W* + *E*(*b*). *W* and *b* are in plaintext and can be modulated for practical and efficient privacy-preserving inference. To help in the implementation of this matrix multiplication with Paillier, which uses positive and integer operands, we make the following adjustments:

- Positive operands: For positive *W* and *b*, we use the in-built training constraints in Keras where the weights and biases are clipped to be greater than or equal to 0. We use Adam optimizer with starting learning rate as 0.0008 with no decay. We monitor the loss value during training and dynamically change the learning rate to accommodate the extra constraints by using callback functions in Keras. We monitor the loss for three iterations before reducing the learning rate by half to a minimum of 0.00001. We also monitor loss for undefined value while training.
- Integer operands: We scale floating point weights to larger integer values for the PHE implementation such that the relative ranking of class probabilities is the same. For security (as discussed in section IV-E), we use key-size = 3072 bits, giving us a large plaintext space to scale the weights. The weights are multiplied by 2^*scale*^ and we choose *scale* = 8 when we get a similar accuracy as with the un-scaled weights.

### C. OPTIMIZATION OF PHE FOR FAST PRIVATE INFERENCE

Here we describe the various optimizations and approximations performed during each phase for the privacy-preserving implementation of the model.

#### 1) ENCRYPTION

We implemented the Paillier cryptosystem using GMP [25], a highly-optimized multiple precision arithmetic library. Paillier requires a random number for encryption, which is raised to the power *N*. This result being *N*-th residue in modulo *N*^2^ corresponds to a ciphertext of zero. Such numbers with initially different randomness can be precomputed and later used to generate new zeros during the computation. This method eliminates the need to raise to *N*-th power whenever a new encryption is required, therefore making computation faster. In addition, based on optimizations described in [26], we precompute the generator *g* raised to all powers of two that are smaller than *N*, thus completely avoiding exponentiation during encryption (exponentiations are replaced by multiplications). We further speed up the encryption process by reducing the number of encryptions. Similar to [27], we pack many individuals into a single ciphertext. This is possible because the plaintext space in Paillier is much larger than what we need for our model for secure key sizes. The process of packing is done in plaintext before encryption using *shift left* and *add* operations. The amount of data that can be packed in one ciphertext is defined by the key size, the scale factor of the weights, and the number of inputs (relevant tag SNPs). Here we represent the inputs with 1 bit and the weights with 8 bits, which leads to an output of 8 + ⌈log_2_ (30)⌉ = 13 bits when selecting 10 tag SNPs as input (30 one-hot-encoded values), while the key size is 3072 bits. Thus, we can pack ⌊3072/13⌋ = 236 values in one ciphertext. Furthermore, our model requires only a subset of tag SNPs, thus, only the information about the relevant tag SNPs is encrypted and forwarded to the query.

#### 2) MATRIX MULTIPLICATION

The query is performed using matrix multiplication, where we multiply an encrypted matrix containing sensitive genotypes of the tag SNPs with a plaintext matrix containing the weights. Since some of the weights may become zero when scaled and converted to integer, we only perform homomorphic operations when the weight is not zero. The homomorphic operations necessary for the matrix multiplication are multiplication of a plaintext by a ciphertext, and addition of ciphertexts. Homomorphic addition of ciphertexts is supported by Paillier, where a modular multiplication of two ciphertexts is equivalent to the addition of the plaintexts. We implemented the multiplication of a plaintext by a ciphertext using additions (similarly to a binary multiplier).

#### 3) DECRYPTION

Decryption is a costly operation, since it contains a modular exponentiation of large numbers. We reduce the number of decryptions by packing as many plaintexts as possible in one ciphertext during encryption, which leads to a packed encrypted output. The packed data is unpacked after decryption using the *shift right* operation and *bitwise and* with a mask. In addition, we added thread-level parallelism to the matrix multiplication (matrix multiplications for different target SNPs are threaded), encryption, and decryption.

### D. ML-BASED GENOTYPE IMPUTATION WITH FHE

In the previous subsections, we discussed several optimizations and approximations to help implementation of private imputation using Paillier cryptosystem. In this sub-section we study the trade-off between security guarantees, accuracy, and performance. As briefly discussed in advantages of FHE, it is quantum resistant, and is currently in the process of being standardized. Further since FHE allows for non-linear operations in encrypted domain (not possible in Paillier), our model selection, and tuning may incorporate complex (unapproximated) operations which may amount to a better accuracy. Thus, for a higher accuracy and better post-quantum security guarantees, we explore a private imputation technique using FHE. Please note, in this exploration, we do not use the approximations in sections III-B and III-C, and use other approximations specific to the FHE scheme.

Wood et. al. survey ML applications for medicine and bioinformatics fields and discuss common solutions for secure GWAS [28]. Logistic regression models and usage of statistical scores like *χ*^2^ have been extensively used in this domain. Furthermore, regression, in general, is widely used to create efficient models for these studies [29–31]. Therefore, to compare with commonly used methods, we built a logistic regression model that prunes features based on mutual information to evaluate efficiency of our methodology. The details of the logistic regression model is described as following.

For each target SNP, we built a logistic regression model that takes the one-hot encoded tag SNPs and outputs a prediction probability given by:

$$P\left(y=k|x\right)=\frac{e^{zk}}{\sum_{l=0}^{2}e^{zl}}$$

where *k* is one of the target genotype [0,1,2], *x* is the tag SNP genotypes, *y* is the predicted target SNP genotype and *z* is the linear combination of weights and biases of the form *Wx* + *b*. Thus, computing a logistic regression model adds one non-linear computation, the type of computation we do not use in our linear models. We experiment with all the tag SNPs to fix inverse regularization factor to 0.1. This parameter decides the penalty of a misprediction. Then we train a logistic regression model on training data for 1,000 iterations using Limited memory–Broyden Fletcher Goldfarb Shanno (LBFGS) optimization algorithm with Python’s scikit-learn library. Please note, the elimination of the non-linear model would have converted it into a linear model, but we evaluate the entire model (including non-linear function) in the encrypted domain to estimate the cost introduced by simple non-linear functions.

We implement the FHE version of logistic regression using TFHE [32], an FHE library that exposes homomorphic gates. TFHE security parameters are left to their default values, which provide 110-bit cryptographic security based on ideal lattice assumptions [33]. The circuits from E3 framework [34] let us abstract the gate logic into optimized arithmetic circuits. With that, we can construct fixed-point arithmetic. Fixed-point additions and subtractions are the same as for the integer type and require only calling the respective circuit. Fixed-point multiplication and division use a homomorphic integer operation and a shift by a constant. Shifting by a constant uses no homomorphic operations, so there is no penalty when compared to the homomorphic integer operation. We also implement a function for homomorphic exponentiation, where the base is a fixed-point ciphertext and the exponent is an integer ciphertext, using squaring and multiplying. Finally, we calculate the exponentiation of the Euler’s constant to a fixed-point ciphertext using Taylor series. Some optimizations, such as pre-calculation of factorials, are applied to that. There have been several approximations of non-linear activations using square functions [35] or piece-wise linear approximation [36]. We choose Taylor series to expand exponentiation for more generic measure of activation. Apart from these approximations, batching can be used to speed-up computations using FHE.

Deeper architectures are able to extract robust features using several layers of various types [37]. Although, deeper architectures will have prohibitive timing overheads (as the number of non-linear functions per target SNP increases), we build deeper architectures to explore the trade-off in accuracy suffered by our linear ML models. Please note that stacking linear layers without non-linear activation functions in between does not constitute a DNN because the linear layers, by property, can be collapsed into a single connection between the input and the output layer. Therefore, for our experiments, we increase the depth of the architecture by creating hidden layers with both linear and non-linear operations. Similar to other models, we use top 10 features using mutual information and build a separate DNN for every target SNP. Each DNN consists of one hidden layer consisting of 16 neurons followed by a sigmoid non-linear activation. We also add a dropout layer while training to remove any co-adaptation of neurons. We use the Adam optimizer with the same learning rate schedule as the linear models to train for 50 iterations reducing categorical cross-entropy loss function. Finally, the hidden layer is connected to the output layer that gives the probability of a target SNP genotype being in one of the classes [0,1,2].

The performance of privacy-preserving ML-models depends on the number of operations in the encrypted domain. Therefore, while a deeper neural architecture with several non-linear activation layers is needed for better accuracy, it also generates high computational overhead.

## IV. EXPERIMENTAL RESULTS

Genotype imputation is essentially a problem of predicting a set of sequences by using another set of sequences as features that we translate into a multi-output multi-class problem to design the neural network architecture. We then develop a privacy-preserving version using Paillier partial homomorphic encryption scheme [17]. The details of our design of neural network architecture, fine-tuning of its parameters, and optimization of Paillier to implement private imputation are discussed in details in Section III.

*Datasets:* We use fully characterized genomes from 2,504 individuals provided by 1000 Genomes Project [22] as our primary dataset. The Chromosome 1 of the human genome is divided into set of tag and target SNPs by iDASH Secure Genome Analysis Challenge’19 [38]. iDASH divided the tag SNPs into two sets. (1) 1k dataset: This dataset contains tag SNPs that are 1kb genomic distance apart from each other, which adds up to a total of 9,746 tag SNPs. (2) 10k dataset: This dataset contains tag SNPs that are 10kb genomic distance apart from each other, which adds up to 1,045 tag SNPs. 1k and 10k refer to the genomic distance, which indicates the number of basepairs between consecutive tag SNPs in the genome. The task is to predict the genotypes of the target SNPs by using the genotypes of either datasets, separately. To design, implement, and evaluate our methodology, we shuffled the dataset and split it into training (80% of individuals) and testing data (20% of individuals). Moreover, for a fair comparison with the state-of-the-art genotype imputation methods, we also use the genotypes of chromosome 1 characterized by whole genome sequencing from 870 individuals of GTEx project (v8) [20] with our model as well as with IMPUTE2 [10] and Beagle [11]. Further, to apply our methodology in a more realistic setting, we impute 80,000 target SNP genotypes by using 16,184 tag SNPs of chromosome 22. The tag SNPs are obtained from Illumina Duo 1M version 3 array platform [39]. We divide the 1000 Genomes dataset into a training set of 1,500 individuals and a test set of 1,000 individuals. We also test this realistic scenario on an independent dataset of 1,927 unrelated individuals from the UK10’s Avon Longitudinal Study of Parents and Children (ALSPAC) project [21]. Summarizing, in our study, we perform four different tests with varied number of individuals and SNPs:

1. Baseline iDASH data (1000 Genomes dataset) of two different genomic distances dubbed 1k dataset and 10k dataset (publicly available toy examples)
2. GTEx dataset as an independent dataset to test our models obtained in (1)
3. 1000 Genomes dataset with a more realistic number of tag and target SNPs (real-world scenario)
4. ALSPAC dataset, as an independent dataset, to test the validity of our model obtained in (3)

### A. PERFORMANCE COMPARISON TO THE STATE-OF-THE-ART METHODS

We implement the designed model in C++, building a class that performs operations in encrypted domain. All experiments are performed using an Intel Xeon Platinum 8259CL 96-core processor with 768 GB RAM running at 2.5 GHz and GMP 6.1.2. For a comprehensive evaluation of the performance of the imputation model, we use a metric that reflects both correct predictions (true positive rate) and false misclassifications (false positive rate) for each of the three genotypes. Therefore, we report the ROC curves, the macro-average accuracy score, the test accuracy, and the micro-average accuracy. The plaintext imputations using Beagle and Impute2 in less than 20 hours [40]. For imputing a maximum of 80,000 targets, we trained 80,000 networks, which approximately took one day running training jobs in parallel. Performance comparison with Impute2 and Beagle excludes training time since it is not a part of online private imputation time. Another reason why our private imputation is relatively faster compared to the plaintext state-of-the-art is because our algorithm skips the phasing step that separates the maternal and paternal haplotypes of a chromosome. Instead, our machine learning based predictions builds model agnostic of which haplotype contains the SNP when the genotypes are heterozygous. Phasing is used for traditional genotype imputation methods and often done using techniques such as Hidden Markov Models and can be time consuming even in plaintext.

#### 1) ACCURACY ON TOY EXAMPLES

**FIGURE 3.**
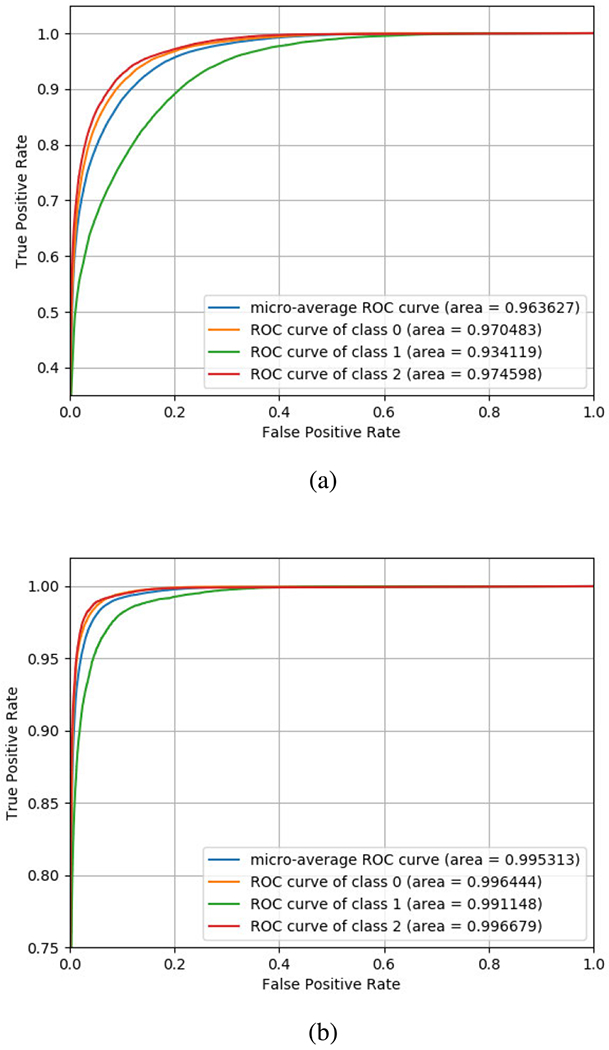
We report the True positive rate with respect to false positive rate (ROC curves) and the micro-average scores on test data for different ML models: (a) Linear neural network model for 10k dataset (b) Linear neural network model for 1k dataset using train-test split of the same dataset for toy examples.

For our baseline dataset, iDASH19 dataset, we plot ROCs for linear models and report the micro-average scores for each in Figs. 3a and 3b for train-test split. For 10k dataset, we achieve 0.9704, 0.9341, and 0.9745 area for classes 0, 1, and 2, respectively, resulting in a micro-average accuracy of 0.9636. For 1k dataset, we achieve a greater 0.99 area for all the three classes with 0.9964, 0.9911, and 0.9966 for classes 0,1, and 2, respectively, resulting in a micro-average score of 0.9953. We achieved a maximum macro-accuracy score of 0.972 on iDASH dataset.

#### 2) ACCURACY ON REAL-WORLD DATA

**FIGURE 4.**
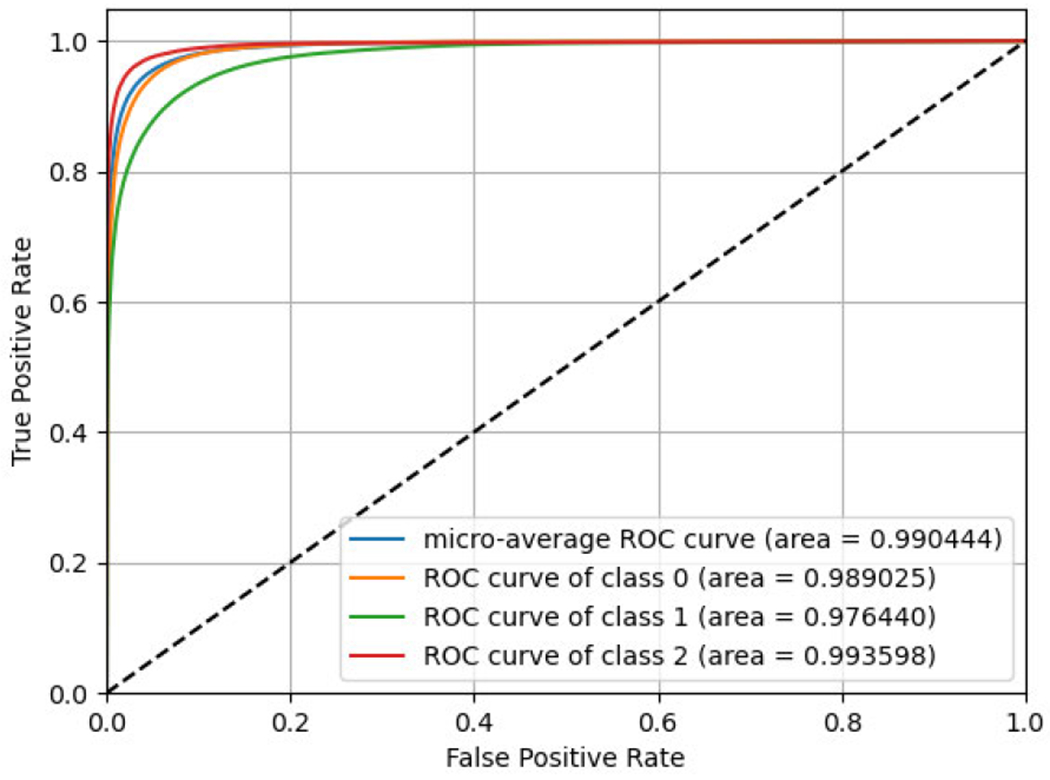
True positive rate with respect to False positive rate are plotted for real-world dataset.

We impute 80,000 target SNP genotypes using 16,184 tag SNPs of chromosome 22 using the same neural network design strategy as followed for iDASH data and report the accuracy in Fig. 4. As mentioned above, these tag and target SNPs are located on human chromosome 22 and obtained from Illumina Duo 1M version 3 array platform [39] We use 1,500 individuals to train and 1,000 individuals to test. We achieve an area under curve of 0.9888, 0.9761, and 0.9935 for classes 0,1, and 2, respectively, resulting in an MAUC score of 0.9903. We also calculate the macro-accuracy and achieve 0.9336 for all variants.

#### 3) TESTING ON INDEPENDENT DATASETS AND COMPARISON AGAINST PLAINTEXT STATE OF THE ART

In this sub-section we further compare our prediction to the results obtained from running commonly used genotype imputation software IMPUTE2 [10] and Beagle [11] using two independent test data from entirely different studies, i.e.GTEx [20] and ALSPAC [21].

##### a: ACCURACY ON GTEx DATASET

We test the accuracy of our toy example model (imputing 500 target SNP genotypes) on GTEx dataset. We find that the performance of our privacy-preserving model is in excellent agreement with the performance of the state-of-the-art plaintext methods such as IMPUTE2 and Beagle as shown in Fig. 6. We achieve micro-average scores of 0.9621 and 0.9946 for 10k and 1k datasets, which correspond to 0.9736 and 0.9959 for Impute2 and 0.9861 and 0.9965 for Beagle.

##### b: ACCURACY ON ALSPAC DATASET

**FIGURE 5.**
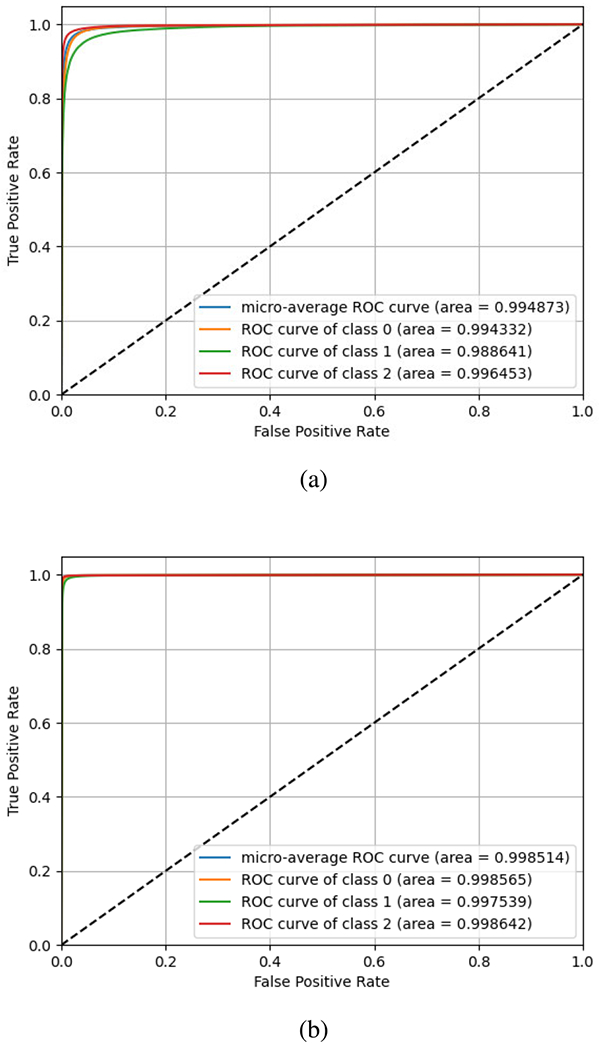
True positive rate with respect to False positive rate are plotted for ALSPAC dataset using (a) our model, (b) Beagle.

**FIGURE 6.**
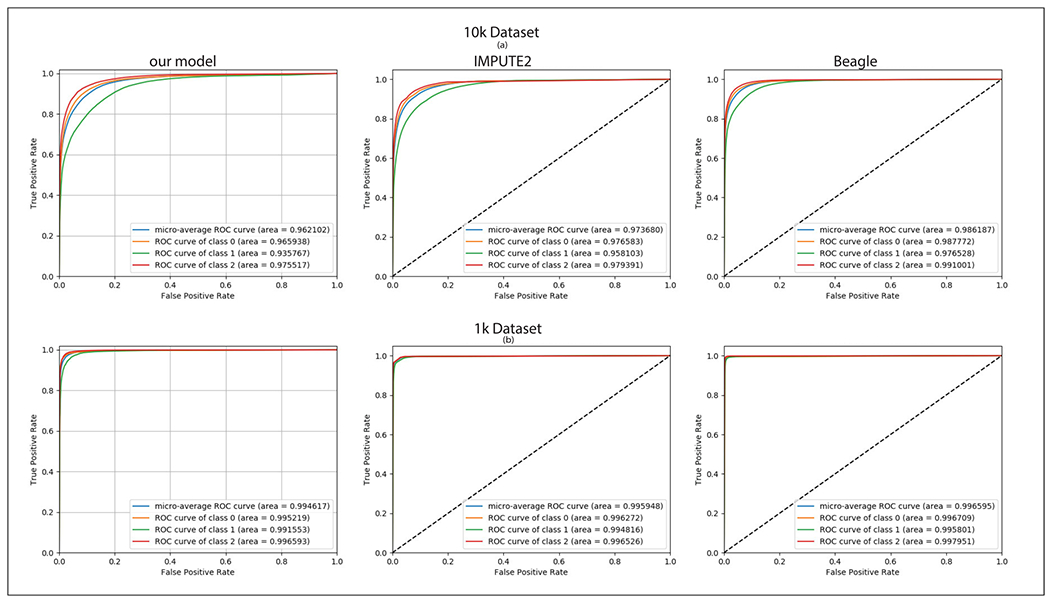
Comparison between our model, IMPUTE2 and Beagle performances. (a) Comparison using the 10k tag SNP data on GTEx individuals. (b) Comparison using the 1k tag SNP data on GTEx individuals.

We further test the accuracy of our real-world model (imputing 80k target SNP genotypes) on ALSPAC dataset. We use the trained model from the real-word data to impute 61,993 target SNPs using 16,184 tag SNPs of chromosome 22 for 1,927 individuals [21]. We achieve a micro-average accuracy of 0.9948 with ROC area of 0.9943, 0.9886, and 0.9964 for classes 0,1, and 2, respectively. This is also in excellent agreement with the performance of the state-of-the-art plaintext method Beagle (Fig. 5b) with a micro-average score of more than 0.99. Since the performance of Impute2 and Beagle are extremely similar in our previous tests, for simplicity, we show here the comparison against Beagle.

Overall, our neural architecture design achieved excellent performance with a varied number of individuals and SNPs on test and independent datasets.

### B. COST EVALUATION: TIMING FOR PRIVATE IMPUTATION

#### 1) PRIVATE IMPUTATION WITH PAILLIER

Encryption takes 0.904 and 0.913 seconds, while decryption takes 2.71 and 2.82 seconds for 10k and 1k datasets, respectively. The fastest part of private imputation is the matrix multiplication, which takes 0.144 and 0.167 seconds, respectively. For both the datasets, private imputation for 501 individuals having 500 target SNPs each, i.e., ≈ 750*K* prediction probabilities, were calculated in under 4 seconds. For real-world dataset, we are able to encrypt the query in 234 seconds, compute in 24.7 seconds and decrypt in 667 seconds. Thus,we perform the entire encrypted imputation for 1,000 individuals in 925.7 seconds for 80,000 target SNPs.

### C. COMPARISON TO OTHER NON-LINEAR ML MODELS

We further develop different non-linear prediction models using FHE to compare against our linear model with PHE.

#### 1) LOGISTIC REGRESSION MODEL

##### a: ACCURACY

We show that private logistic regression with FHE achieves a test accuracy of 86.07% and 96.22% for the 10k and 1k datasets, respectively. We found that the micro-average scores to be 0.9704 and 0.9967 for 10k and 1k datasets, respectively. We also observe that the micro-average scores of our linear model and the non-linear logistic regression model differ by less than 0.01. Therefore, the approximations done to our model are able to achieve accuracy close to the non-linear models for a tremendous improvement in speed.

##### b: COST

The logistic regression models for both 1k and 10k use the same number of features as our linear model. Therefore, here we report the computation time for 10k dataset for different bit sizes in Table 2. We remark that each bit is a different ciphertext. Using the smallest (fastest) non-linear model, we perform private imputation for one individual in ≈ 3 hours and ≈ 19 hours with 8-bit and 16-bit precision, respectively. Our model was run using a single thread, thus scaling it to multiple threads would improve performance by 2 orders of magnitude. But even then, the linear model using Paillier is 7 orders of magnitude faster.

#### 2) DEEP NEURAL NETWORKS (DNN)

##### a: ACCURACY

We show that private deep neural networks achieves an accuracy of 85.74% and 96.05% and 0.9688 and 0.9964 MAUC for 10k and 1k datasets, respectively. This shows an approximately 1% increase in accuracy compared to our linear model. The ≈ 1% increase in accuracy and ≈ 0.002 – 0.006 increase in MAUC scores using a deeper architecture is intuitive as deeper models extract more relevant features. But considering the trade-off between the increase in accuracy and impractical computational overheads, we think that the linear models provide sustainable performance.

##### b: COST

Similar to our logistic regression model that has one non-linear function, our non-linear DNN also has one non-linear function in the hidden layer. Also, both the logistic regression models and the DNNs have exponentiation in non-linear activation. Therefore the computation for the non-linear operation may be estimated to be similar to logistic regression model. Additionally, the model computes matrix multiplication (linear operation) for one hidden layer, which results in further overheads. Having similar estimated timings costs, any DNN will be at least 7 orders of magnitude slower than our Paillier linear model.

### D. SCALABILITY

The accuracy of an ML model depends on the processing of the input feature map, and the cost of operation of the model depends on the number of outputs being predicted. Therefore, for the scalability study, we analyze the variation in accuracy with the increasing number of tag SNPs (inputs) and the variation in costs as a function of number of target SNPs being predicted.

#### 1) ACCURACY

To validate the scalability of our approach for private imputation, we primarily test it across datasets and state-of-the-art genotype imputation tools as explained in the IV-A section. In this sub-section, we validate scalability across genomic distances between tag SNPs from 1k to 10k in terms of accuracy.

**FIGURE 7.**
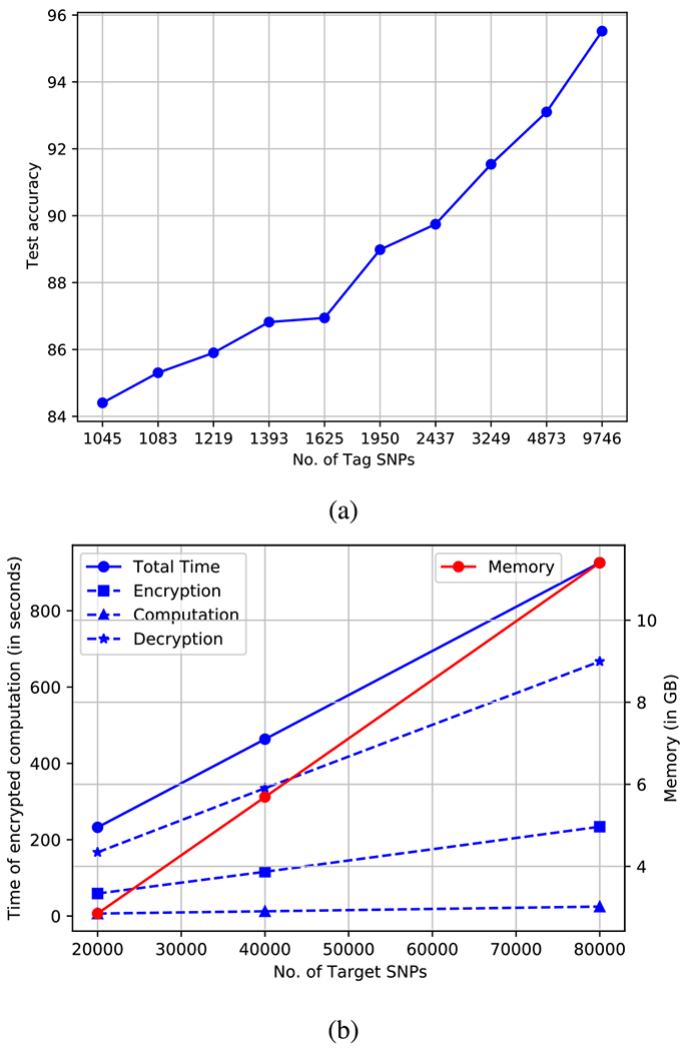
(a) Variation of test accuracy as a function of number of tag SNPs (as the genomic distance is reduced in intervals of 1kb distance). The plot shows an increase in test accuracy as more number of tag SNPs become available to choose from. (b) Variation in time and memory requirements as a function of number of individuals queried in encrypted domain.

We implement linear models by using a top 10 tag SNPs through mutual information per target SNP. The tag SNPs used for mutual information calculation were selected based on different genomic distances between them. We report the change in accuracy as a function of available tag SNPs (as genomic distance is reduced) in Fig. 7a. We observe a gradual linear increase in accuracy as the genomic distance between tag SNPs is decreased (i.e. as more tag SNPs are available). This is intuitive since more tag SNPs are available to choose from. We observe that our approach is consistent across datasets when tag SNPs are taken based on different genomic distances between them.

#### 2) TIME AND MEMORY REQUIREMENTS

We take the first 20,000, 40,000, 80,000 SNPs of human chromosome 22 using 1,500 individuals as training and 1,000 individuals as a test set from 1000 Genomes dataset to perform imputation and report the costs in Fig. 7b to study scalability in timing and memory. We observe that the costs scale linearly as a function of target SNPs being imputed. We observe that the rate of increase in decryption time is larger than the rate of increase in encryption time, while the rate of increase in computation time is the lowest. From the figure we observe a linear increase in costs and that 80,000 targets for 1,000 individuals can be imputed in 925.7 seconds. Using these values, we find that 1 million target SNPs can be imputed in approximately 3 hours. Further, with our optimized implementation, the speed can further be increased by deploying more threads and cores. The memory requirements are also practical considering 80K SNPs were imputed using 11.4 GB memory.

### E. SECURITY DISCUSSION

The security of our implementation of private imputation is solely dependent on the security of Paillier PHE which is based on the decisional composite residuosity assumption and factorization hard mathematical problems [17]. According to NIST guidelines on key management [41], *N* = 3072 bits ensures 128-bit security in symmetric key cryptography domain which has been standardized to be secure.

## V. DISCUSSION

In this work, we present a privacy-preserving genotype imputation methodology using the Paillier cryptosystem, a standardized partial homomorphic encryption scheme. But since Paillier is restricted by the types of operations it can perform, we make several approximations in our plaintext methodology and optimizations in its Paillier implementation for standardized privacy-preserving imputation. For evaluation of our final plaintext methodology, we compare its performance with other plaintext state-of-the-art solutions as well as other complex non-linear models. For 10k dataset, we observe that the MAUC scores of IMPUTE2 and Beagle are just 0.0115 and 0.0240 more than our models’ MAUC scores. For 1k dataset, the difference in MAUC score is even lower, being 0.0013, and 0.0019, for IMPUTE2 and Beagle, respectively. Comparing with non-linear logistic regression model, we find our model’s MAUC scores are just 0.0068 and 0.0014 lower for 10k and 1k datasets respectively. But adding one non-linear function to the model increases the time of computation by several orders for this slight increase in accuracy. Further, we test our models on independent datasets GTEx, and ALSPAC, and achieve a score of 0.9904 and 0.9948, respectively which is similar to the scores achieved by state-of-the-art imputation tools. Therefore, our approximations in the ML model and optimizations in the implementation of Paillier lead to a tremendous improvement in the performance for a slight reduction in accuracy/MAUC score.

One of the key properties of our imputation method is that it does not require phasing the genome into haplotypes, which allows us to perform the imputation with a fairly less computational cost. On the other hand, although our model makes accurate genotype imputation for all genotype classes, we observe a slight decrease in accuracy when we impute heterozygous genotypes. We think that since our model is “phasing free”, determining the correlations between the target SNP genotype and tag SNP genotypes in a haplotype is difficult when the genotype is heterozygous. In linkage disequilibrium, one expects to see the existence of a genotype being correlated with the existence of other genotypes in the same haplotype, which requires the knowledge of which haplotype has the target and tag SNPs. When the genome is not phased, this information is lost, hence the correlation between genotypes in the same haplotype cannot be established correctly. We think that since the predictions are good even for heterozygous genotypes, skipping phasing is an adequate trade-off in terms of computational cost.

## VI. CONCLUSION

In this study, we show that complex algorithms could be manipulated and used in Paillier cryptosystem of PHE in a computationally effective and biologically accurate manner in the inference time. Using several optimizations and approximations we were able to perform imputation for 80K SNPs in practical time whilst maintaining imputation efficacy of above 0.99 MAUC score. For future work we want to explore methodologies like federated learning to perform training of the model in a privacy-preserving manner to make the entire pipeline private. We envision that improvements made in this study can be used in other large-scale privacy preserving genome analysis tool development (e.g. haplotype phasing).

## Figures and Tables

**TABLE 1.** Time cost of genotype imputation model for 10k and 1k dataset each with top 10 features (i.e. top 10 tag SNP genotypes).

Operation	Timing (in seconds)	
	10k Dataset	1k Dataset
Encryption	0.904	0.913
Computation	0.144	0.167
Decryption	2.71	2.82
Total	3.758	3.9

**TABLE 2.** Performance of Logistic regression model using FHE for 1 target SNP and 1 individual.

Operation (in seconds)	Size (8 bits)	Size (16 bits)
Matrix multiplication	8022.05	41513.2
Bias addition	13.67	29.53
Exponentiation	2844.45	27806.5

## Data Availability

**Code:** https://github.com/momalab/octal-impute Data: iDASH challenge 2019 Track II data: https://github.com/momalab/octal-impute Real-world dataset: https://support.illumina.com/downloads/human1m-duo_v3-0_product_files.html ALSPAC and GTEx datasets are not publicly available but available upon approval to access in EGA and dbGAP, respectively.
